# Illinois State Medical Society

**Published:** 1855-07

**Authors:** 


*Illinois State Medical Society.*

This Society held its regular annual meeting on the first Tuesday and Wednesday of June, at Bloomington. About forty members were present, the greater part of whom were from the northern and middle sections of the State. At 10 o'clock A.M., on Tuesday, the society was called to order by Dr. C. N. Andrews, the President; and Dr. A. H. Luce, chairman of the Committee of Arrangements, reported the names of those delegates who had presented their credentials. After the usual preliminary business, the following officers were elected for the ensuing year, viz.:

PRESIDENT:

DR. N. S. DAVIS, of Chicago.

VICE-PRESIDENTS:

Drs. F. A. McNEIL, of Mount Morris, and H. NOBLE, of Leroy.

SECRETARIES:

Drs. A. H. LUCE, of Bloomington, and E. ANDREW, of Peoria.

TREASURER:

Dr. J. V. Z. BLANEY, of Chicago.

During the continuance of the annual session, reports were made by the Treasurer and Committee on Publication; by Dr. J. W. Freer, of Chicago, chairman of the Standing Committee on Surgery; by Dr. E. Roe, of Bloomington, chairman of the committee on Practical Medicine; and by Dr. H. A. Johnson, of Chicago, chairman of the Committee on Drugs and Medicines.

The report of Dr. Roe was chiefly occupied with the consideration of Typhoid Fever, and the efficacy of certain remedies in its treatment.

The reporter advocated the doctrine, that a very moderate alterative influence (or "slight salivation") from mercury was decidedly beneficial in the early stage of this variety of fever, and in many instances capable of arresting its progress. This part of the report led to a very interesting discussion, which was participated in by Dr. Roe; Drs Andrews and Clark, of Rockford; Dr

Prince, of Jacksonville; Dr. Rogers, of Peoria Co.; Drs. Bailey and McArthur, of Joliet; Dr. McNeil, of Mount Morris; and Drs. Herrick and Davis, of Chicago. As might be expected ,much diversity of sentiment was expressed in reference both to the nature and treatment of continued fevers. Only a few agreed fully with the chairman of the Committee in reference to the value of mercurials in this form of disease, while it was plainly evident from the whole discussion, that grades of fever widely diverse in the symptoms presented, in the tendencies observed, and in the results b-tained, have been grouped under the rather popular name of Typhoid Fever. It might almost be said that it had become fashionable to dignify every case of febrile disease, not profound typhus on the one hand nor a plain ague on the other, with the name of Typhoid Fever. Hence we not unfrequently have a protracted enteric fever and the simplest ephemera ranked in the same class. It is evident therefore, that until a more definite rule of diagnosis is established and generally adhered to, it will be vain to look for any approach towards uniformity of views in regard to the treatment of this important class of diseases.

Though that part of the report by Dr. Roe, relating to the prevalence of epidemics in the State, during the past year, was very defective, yet both the report and the discussion to which it gave rise, were highly interesting and profitable.

The report of the committee on Drugs and Medicines, was also listened to with much interest; and an allusion in it to the use of Iodine as an antidote to the poison of serpents, and the probability of its being equally capable of destroying all the animal poisons, led to the relation of some interesting facts by Dr. A. L. McArthur of Joliet.

He stated that by a Railroad accident near Joliet during the past year several persons were very severely burned. Some of these burns resulted in the formation of extensive suppurating ulcers, coupled with general typhoid symptoms. In several instances the pus from these ulcers proved highly poisonous, inoculating the hands of those who dressed the surfaces wherever there happened to be the slightest abrasion of the skin. The symptoms produced by such inoculation, were those of a severe phlegmonous erysipelas. In these cases the application of the solution of Iodine seemed to be promptly beneficial in destroying the poison. Dr. F. K. Bailey, witnessed some of the same cases, and coincided with the statements of Dr. McArthur. Dr. Roe, of Bloomington, expressed the opinion that Iodine is an effectual antidote to the poison both of syphilis and erysipelas.

On the afternoon of the second day, the retiring president, Dr. C.N.Andrews, of Rockford, delivered his valedictory Address. The subject was, "Medical Progress;" and it was treated in such a manner as to indicate a commendable degree of industry and research on the part of the author. We shall defer any analysis of it until we have it in printed form before us. The usual standing committees were appointed on Practical Medicine, Surgery, Obstetrics, and on Drugs and Medicines. Besides these, there were appointed several special committees, to each of whom was assigned a specific subject for investigation; which will doubtless add much to the interest of the next annual meeting. A committee consisting of Drs. D. Prince, of Jacksonville, J. V. Z. Blaney, of Chicago, and E. Roe, of Bloomington, was appointed to memorialize the next Legislature in favor of providing additional accommodations for the Insane, and for the instruction of imbecile and idiotic children.

Drs. W. B. Herrick, of Chicago, R. Rouse, of Peoria, and F. A. McNeill, of Mt. Morris, were appointed a committee on prize essays, with instruction to offer a premium of fifty dollars for the best essay on some medical subject. Essays designed as competitors for this prize, should be sent to the chairman of the committee on or before the first of April next. Twelve delegates were appointed to attend the next annual meeting of the American Medical Association.

The meeting throughout was characterized by an untiring devotion to its legitimate business, by the most perfect good feeling and social harmony, and will doubtless be remembered with pleasure by all who attended. Vandalia was selected as the place for the next annual meeting, which will be held on the first Tuesday in June, 1856.

